# Association between *IL6 *gene polymorphism and the risk of chronic obstructive pulmonary disease in the north Indian population

**DOI:** 10.22099/mbrc.2019.34594.1431

**Published:** 2020-06

**Authors:** Nikhil Kirtipal, Hitender Thakur, Ranbir Chander Sobti, Ashok Kumar Janmeja

**Affiliations:** 1 Department of Biotechnology, Panjab University, Chandigarh-160014, India; 2 Assistant Professor, Dept. of Biotechnology, SUSCET, Tangori, Punjab-140306, India; 3 Respiratory Medicine, MMCH, Solan, Himachal Pradesh, India

**Keywords:** Chronic obstructive pulmonary disease, Interleukin-6, Genotypes, Cytokine

## Abstract

Interleukin-6 (IL6) is encoded by the *IL6* gene in human and acts as pro-inflammatory cytokine and an anti-inflammatory cytokine. Recent studies established that *IL6* substantially contribute in the diagnosed of systemic inﬂammation for the patients suffering from lung diseases such as chronic obstructive pulmonary disease (COPD). Thereof, this work aimed to investigate the protagonist of *IL6* (-174 G/C) genotypes as an essential risk factor for COPD in north Indian population. In the study, a total of 200 clinically diagnosed patients with COPD were selected against 200 patients. Statistical analysis reveleaed that there was no significant association between the IL6 -174 G/C genetic polymorphism and the risk of COPD (P>0.05).

## INTRODUCTION

Chronic obstructive pulmonary disease (COPD) has been categorised as systemic disease and characterized for narrowing or obstruction of airways and resulted into chronic bronchitis or emphysema. It has been also considered with abnormal inflammation in the respiratory tract and lung by noxious particles or gases [1]. Generally, smoking has been suggested as major factor for inducing COPD risk but specific mechanism for the pathogenesis is not adequately studied [2]. Recent studies suggested that individuals diagnosed for only chronic bronchitis with no substantive airflow limitations should not be characterized as COPD 3]. Moreover, recent investigations also established the COPD association with smoking as symptomatic COPD development in 50% smokers [4]. 

Cytokines are major components for chronic inflammation in all the diseases as well as in COPD [5]. It was reported that *IL6* gene (MIM:147620) plays an active role in pathogenesis of lung disease like asthma [6]. Hence, *IL6* gene may be a relevant and appropriate target for the treatment of COPD and other associated chronic lung diseases [7]. The present study was conducted to establish the relationship between *IL6* (-174 G/C) polymorphism and risk of COPD in a population from the northern region part of India. 

## MATERIALS AND METHODS

This study included 200 (114 males, 86 females) COPD patients and 200 (134 males, 66 females) healthy blood donors as controls. The patients and ontrols were recruited for the study as described previously [8]. Genotyping of the *IL6* (-174 G/C) polymorphism was carried out as reported previously [9]. The statistical relevance of relationship between genotypes and risk of COPD was defined by odds ratio (OR) with its 95% confidence interval (95% CI). Statistical analyses were carried out using the statistical packages of SPSS software version 11.5. 

## RESULTS AND DISCUSSION

Table 1 show the genotypes of the participants in respect with their smoking ahbit. Genotypic frequency in control group showed no variation from the expected values based on the Hardy-Weinberg equilibrium. The statiscical analysis reveleaed that there was no significant association between the IL6 -174 G/C genetic polymorphism and the risk of COPD (Table 1).

**Table 1:** Association between the *IL6* gene (-174 G/C) polymorphism and risk of COPD

**Genotypes**	**Cases (200)**	**Controls (200)**	**OR (95% CI)**	**P**
Total				
GG	90	105	1.0	-
GC	85	78	1.27 (0.82-1.97)	0.306
CC	25	17	1.72 (0.82-3.57)	0.160
				
Smokers				
GG	56	56	1.0	-
GC	52	34	1.53 (0.83-2.81)	0.186
CC	18	11	1.64 (0.660-4.11)	0.341
				
Non-smokers				
GG	34	49	1.0	-
GC	33	44	1.08 (0.55-2.13)	0.934
CC	7	6	1.68 (0.45-6.30)	0.567

COPD is a complex inflammatory lung disease which includes the characters of emphysema, chronic bronchitis and asthma in patients. Although, many inflammatory cells, mediators and enzymes have been established for COPD development, but their importance is not yet well understood. This study was carried to understand the relationship between *IL6 *polymorphism and COPD risk in cigarette smokers of north Indian population. Statistical analysis suggested that *IL6* (-174G/C) polymorphisms had no association with COPD risk. Similar finding was conducted on 243 individuals (including 113 COPD cases) to elucidate the association between -174 (G/C) polymorphism of the *IL6* promoter gene and COPD in Caucasian German populations, concluded no correlation between them [10]. The -597G/A and -174G/C polymorphisms in *IL6* gene was also reported with no association with COPD risk in Spanish population [11]. Likewise, present study was conducted with an equal number of COPD and control patients, which was larger in population size, also predicted no significant association between *IL6* genotypes and COPD development. Since, a limited number of studies on relationship between *IL6 *(-174) polymorphism and COPD were reported so far, furthermore evaluations with large population may require for clarifying the relation of *IL6 *(-174) polymorphism and COPD.
